# An easy-to-use primer design tool to address paralogous loci and T-DNA insertion sites in the genome of *Arabidopsis thaliana*

**DOI:** 10.1186/1746-4811-10-28

**Published:** 2014-09-13

**Authors:** Gunnar Huep, Nils Kleinboelting, Bernd Weisshaar

**Affiliations:** 1 Center for Biotechnology & Department of Biology, Bielefeld University, Universitaetsstrasse 25, D-33615 Bielefeld, Germany

**Keywords:** *Arabidopsis thaliana*, T-DNA, Insertion mutants, Paralog, Primer design, GABI-Kat

## Abstract

**Background:**

More than 90% of the *Arabidopsis thaliana* genes are members of multigene families. DNA sequence similarities present in such related genes can cause trouble, e.g. when molecularly analysing mutant alleles of these genes. Also, flanking-sequence-tag (FST) based predictions of T-DNA insertion positions are often located within paralogous regions of the genome. In such cases, the prediction of the correct insertion site must include careful sequence analyses on the one hand and a paralog specific primer design for experimental confirmation of the prediction on the other hand.

**Results:**

GABI-Kat is a large *A. thaliana* insertion line resource, which uses in-house confirmation to provide highly reliable access to T-DNA insertion alleles. To offer trustworthy mutant alleles of paralogous loci, we considered multiple insertion site predictions for single FSTs and implemented this 1-to-N relation in our database. The resulting paralogous predictions were addressed experimentally and the correct insertion locus was identified in most cases, including cases in which there were multiple predictions with identical prediction scores. A newly developed primer design tool that takes paralogous regions into account was developed to streamline the confirmation process for paralogs. The tool is suitable for all parts of the genome and is freely available at the GABI-Kat website. Although the tool was initially designed for the analysis of T-DNA insertion mutants, it can be used for any experiment that requires locus-specific primers for the *A. thaliana* genome. It is easy to use and also able to design amplimers with two genome-specific primers as required for genotyping segregating families of insertion mutants when looking for homozygous offspring.

**Conclusions:**

The paralog-aware confirmation process significantly improved the reliability of the insertion site assignment when paralogous regions of the genome were affected. An automatic online primer design tool that incorporates experience from the in-house confirmation of T-DNA insertion lines has been made available. It provides easy access to primers for the analysis of T-DNA insertion alleles, but it is also beneficial for other applications as well.

## Background

*Arabidopsis thaliana* is widely and very successfully used as a model organism in basic plant research. After the completion of its genome sequence in the year 2000 [1], several large mutant collections have been established. In most cases, T-DNA insertional mutagenesis mediated by *Agrobacterium tumefaciens* has been used for the generation of knock out alleles for reverse genetic approaches [2,3]. The insertion of T-DNA in the plant genome occurs almost randomly [4-6], and different methods for the identification of insertion sites in specific lines have been established. The most frequently used method is based upon flanking sequence tags (FSTs). FSTs are short DNA sequences, which flank the T-DNA insertion site and contain genome sequence information adjacent to the insertion site. They are generated with PCR-based methods after digestion of genomic DNA and adapter ligation [7]. Their sequences can be compared to the *A. thaliana* genome sequence using BLAST [8] to predict the insertion position(s) of the T-DNA in a given line. The GABI-Kat collection is the world’s second largest FST-based T-DNA insertion line collection for *A. thaliana*[9]. The FST data along with insertion site predictions and information about confirmed T-DNA insertion alleles is accessible at SimpleSearch, which is the user interface to the database at the website of the project [10]. GABI-Kat lines can be accessed via SimpleSearch, and links to the stock centres are provided if the line that contains the relevant allele has been donated to the American and/or European stock centres for *A. thaliana* seeds [11]. In case of direct orders at GABI-Kat, predicted insertions are requested rather than simply seed of GABI-Kat lines. Upon a user request, T2 plants of the respective line are grown and the insertion site prediction is confirmed at GABI-Kat by PCR with an insertion site-specific primer and a T-DNA border primer, followed by sequencing of the amplicon. The experimental data for confirmed insertion alleles is presented on the SimpleSearch website [12]. This includes the amplicon sequences as well as the sequences of the primers used in the confirmation PCR. A more detailed overview on the features of the SimpleSearch site is summarised in [9].

A major problem during the FST-based insertion site prediction in T-DNA insertion lines occurs when the FST sequence cannot be assigned unambiguously to a *single* specific locus in the *A. thaliana* genome. Such events are inevitable, because even in a small genome like the one from *A. thaliana* only about 10% of all genes encode unique proteins. All other genes have at least one additional homologue [13,14]. One reason for the occurrence of homologues within the genomes of eukaryotes lies in genome duplication events leading to paralogous genes. This has already been studied in detail in the original *A. thaliana* genome sequence analysis [1], and searchable databases are available which support analyses of duplication events and paralogous gene families [15]. Beside genes (and without considering transposable elements), also non-genic sequences of the *A. thaliana* genome occur in higher copy numbers. Several mechanisms have been discussed for the duplication events (reviewed for example in [16]). Regardless of the exact mechanism, the ultimate result of duplication events is that even after evolutionary diversification of the duplicated sequences, larger stretches of similar sequences occur at different positions in the *A. thaliana* genome. In this article we will refer to regions with more than one copy of similar DNA sequences in the genome as “paralogous regions”, regardless if genic or non-genic regions are concerned. In this sense, we will also use the term “paralog” for individual regions in paralogous regions, even if those regions are non-genic and if the genetic origin (i.e. duplication event) of the respective region is not clear.

In all large FST-based T-DNA insertion line collections, the FSTs have so far been used to predict a single locus in the genome as the corresponding insertion site. If the locus is located within a paralogous region, the prediction and the decision for one of the paralogs is error-prone. However, given that the sequences of the paralogous loci are known, the confirmation process at GABI-Kat, which considers the DNA sequence of the confirmation amplicon, is able to resolve ambiguities concerning the correct insertion position in most cases. Only if the paralogous regions contain (almost) identical sequences a definite assignment to a single locus is not possible.

We present data from example cases in which the correct insertion locus was identified only after PCR-based confirmation using optimised primers, even though FST-based insertion site prediction was unable to assign a unique best-fitting locus. Insertion site predictions were redone using the TAIRv10 genome sequence and BLAST, and multiple predictions derived from single FSTs were combined into “paralog groups”. When attempting to confirm a prediction from such a group, specific primers (as far as possible) unique for relevant insertion sites were designed. We developed a primer design method that identifies possible primers using a multiple alignment, which enables the discrimination between the different paralogous regions. An optimised, easy to use version of the tool is available on the website of GABI-Kat and allows users to design primers at their own locus or genome position of interest.

## Results and discussion

### FSTs and T-DNA insertion site predictions

The GABI-Kat database contains insertion site predictions from about 135,000 FSTs, which were generated for the 93,504 lines in the T-DNA insertion line collection. During the generation of GABI-Kat FSTs, genomic DNA of individual T1 plants was digested with *Bfa*I, adaptors were added, and fragments containing T-DNA borders as well as sequences of plant origin next to the T-DNA were amplified with a T-DNA- and an adaptor-specific primer [7]. The length of the resulting amplicons is dependent on the position of the *Bfa*I recognition sites in the genome relative to the insertion site. In case of more than one T-DNA insertion in a given line, more than one amplicon might be generated in a single reaction. Due to different sizes of these amplicons, an insertion corresponding to a longer amplicon is measured at the tail of the FST sequence, and an insertion corresponding to a shorter amplicon at the head of the FST sequence. Usually the shorter amplicon causes a stronger signal because of higher fragment abundance after PCR. We refer to these FSTs, which allow to correctly predict several insertion sites in one line from different regions of one FST, as “composite FSTs”. Such FSTs have also been described for the SALK collection [17].

We often observed that GABI-Kat FSTs contain sequence parts from borders of two distinct T-DNA insertions present in a single line. When addressing insertion site predictions in paralogous regions of the genome, predictions from “composite FSTs” had to be considered as well because they share the feature “additional BLAST hit from one FST”. The optimised analysis pipeline (see below) that has been established at GABI-Kat detects, in addition to paralogous hits, also hits from “composite FSTs”. The additional predictions derived from GABI-Kat FSTs by using this optimised pipeline have been made available with the GABI-Kat database release No 27 [12].

Initially, the insertion predictions were deduced from the FSTs in a 1-to-1 relation. Only the best BLAST hit from a given single FST was evaluated for the prediction of a single insertion site [9,18]. In order to address paralogous regions of the genome, we recalculated the insertion site predictions for all FSTs. For this new assessment, a 1-to-N relation of FST to insertion site predictions was implemented in the internal GABI-Kat database. To be able to filter for the most relevant predictions, three categories (designated 0, 1 and 2) were defined and assigned to the different types of insertion predictions deduced from a single FST based on the BLAST e-value. Category 0 was assigned to the prediction deduced from the best BLAST hit. This was the one that had been selected as the only prediction (1-to-1) before the extended analysis (1-to-N) was performed. Additional predictions from the evaluated FST were assigned to category 1 if the BLAST e-values were lower than 1e-3, and to category 2 if the e-values were 1e-3 or higher (for details see Methods). The additional predictions were taken into account during the confirmation process at GABI-Kat if necessary. This was especially important if the e-values of the BLAST hits for a given FST region were highly similar or even identical because paralogous genomic loci were affected. Only one of the insertion site predictions derived from a single region of an FST corresponds to the correct insertion locus. Details about the results from the “1-to-N” type FST evaluation and insertion site prediction are listed in Table S1, which is included in the document “Additional file 1”.

We observed that the prediction of category 0 could be wrong due to small errors in the FST sequence, even if the BLAST analysis results in a unique best hit. Consequently, analysis of only this locus would have made confirmation impossible. The access to several BLAST hits from one FST region allowed creating groups of paralogous insertion site predictions of categories 0 and 1, which were derived from subsets of the FSTs of the respective line. In total, about 11,000 paralog groups were detected in the GABI-Kat FST dataset. If a paralogous prediction was addressed for confirmation experimentally, several predictions in the respective group were analysed during the confirmation process, if necessary. Until now, more than 1,200 groups with paralogous predictions in the GABI-Kat collection have been solved experimentally. If a prediction other than the best prediction has been confirmed, this prediction was made available in SimpleSearch in addition to the “category 0 prediction” which was included anyway.

### Primers in paralogous regions of the genome

Even when most parts of paralogous sequences are highly similar or even identical, the individual sequences often differ at certain positions. Based upon sequence alignments of the genomic DNA sequences of the individual paralogs in groups of paralogous predictions, we developed a primer design algorithm that allows designing specific primers (see Methods). Uniqueness for the individual paralog was preferably constructed into their 3′-ends. Such primers can enable the determination of the correct insertion site prediction by PCR and sequencing, even if only one base pair differs in the paralogous regions surrounding the set of paralogous insertion site predictions. A direct comparison of PCR results with the different paralog-specific primers allows the discrimination between the paralogs, sometimes taking into account that mispriming usually leads to weaker PCR products.

In addition to paralogous regions in the genome, random mispriming sites in the genome might occur for primers. In our experience and with our PCR conditions, even short sequence stretches at the 3′-end of primers can lead to unspecific PCR products (see [19] and references therein). We have regularly observed examples of primers, which were able to amplify unspecific PCR products when only 12 bases of their 3′-end had a perfect match in the genome. For example, in GABI-Kat line 011B05 we tried to confirm the predicted insertion at position 51,137 on chromosome 3. The primer that was used for this purpose (5′-CTCAATTTATGTGTGACTGCAAGC-3′) had the unique, perfect annealing site from position 50,794 to 50,817 on chromosome 3. Unexpectedly, we observed an amplicon of roughly 1.3 kb. BLAST analysis of the sequence of this amplicon resulted in a hit in the gene *At4g33170* with a BLAST e-value of 0.0 and a derived T-DNA insertion site at position 15,997,766 on chromosome 4. Analysis of the primer sequence showed a perfect match of the last 12 bp at the positions 15,996,481 to 15,996,492 on chromosome 4. The insertion in the line 011B05 was subsequently confirmed with an *At4g33170*-specific primer, essentially by using the “wrong” confirmation sequence as an FST for insertion site prediction. More extreme examples of mispriming occur in rare cases. This is taken into account in our primer design by minimizing the number of possible 12 bp-matches within the genome (see Methods).

### Application of the paralog primer method

**Figure 1:**
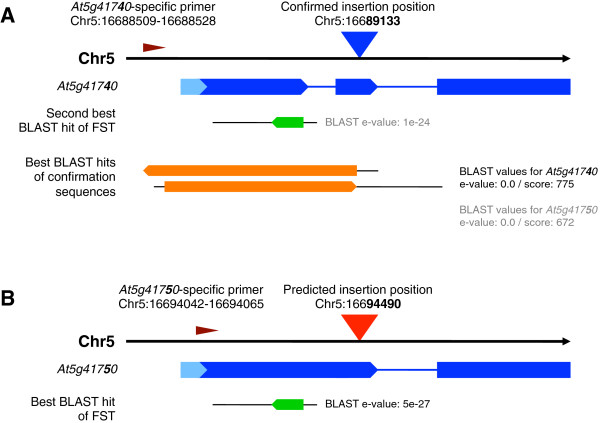
**Confirmation of the insertion allele of *At5g41740 *in the GABI-Kat line 683F05.** In line 683F05 two paralogous loci were predicted as possible insertion sites. Initiated by a user request for *At5g41750*, both loci were examined with primers specific for the respective paralogous locus. As a result, the worse prediction could be confirmed successfully **(A)** and the PCR for the better prediction failed **(B)**. FST-based insertion site predictions are shown with blue (confirmed) and red (failed) triangles. Primer positions are given using dark red arrowheads. The affected genes are symbolised by blue arrows. Light blue arrows are 5′-UTRs, CDSs are shown in dark blue. Introns are indicated by thin blue lines. BLAST hits of the FSTs are shown as green arrows, BLAST hits of the confirmation sequences are shown as orange arrows. Sequence parts of FSTs and confirmation sequences that do not fit to the indicated positions are indicated as thin black lines. The genomic positions in the figure are given according to the TAIRv10 genome sequence dataset of *A. thaliana*.

An example that illustrates the advantages of the paralog primer method is the confirmation of the insertion in *At5g41740* in the GABI-Kat line 683F05 (see Figure 1). The best insertion site prediction and the only one that had been available in the “1-to-1 prediction dataset” in this line was *At5g41750* with a BLAST e-value of 5e-27. This gene is annotated to encode a “disease resistance protein (TIR-NBS-LRR class) family” in TAIRv10. After newly calculating the insertion site predictions and setting up the “paralog groups”, the second best prediction for the same FST was *At5g41740* with a BLAST e-value of 1e-24 and the same annotation. The experimental analysis with paralog specific primers designed using the tools described above resulted in an amplicon with the *At5g41740*-specific primer and no product with the At5g41750-specific primer. BLAST analysis of the sequence of the confirmation amplicon resulted in fully aligning sequences for both genes. However, when comparing the score values, *At5g41740* reached 775 while *At5g41750* ended up with 672. Manual inspection of the alignments confirmed a few SNP positions that distinguish the two loci with *At5g41740* representing the correct locus. The confirmed insertion in *At5g41740* is available in SimpleSearch with a search for the respective AGI gene/locus code, and the data from the experimental analysis as well as a link to the stock centre NASC are displayed.

Another example is the confirmation of the insertion in *At1g07930* in the line 902F05. In this line the best predictions were *At1g07920* and *At1g07940* with a BLAST e-value of 1e-95 in both cases. A third and slightly worse prediction for the same FST was *At1g07930* with a BLAST e-value of 7e-91. Only for *At1g07930* a PCR product could be obtained with paralog-specific primers. BLAST analysis of the sequence of the amplicon confirmed *At1g07930* as the correct paralog via the score values similar to the example above (data available at the SimpleSearch website for line 902F05).

Besides examples of GABI-Kat lines with second- or third-best insertion site predictions being confirmed, there are several cases of lines which have two or more predictions with identical reliability according to the BLAST e-values of the FSTs. Primers designed using the tools described above allowed the determination of the correct insertion locus in these lines. Examples are the GABI-Kat lines 583H04 and 742E06. In line 583H04, *At1g29350* was identified as a wrong prediction and the insertion in *At1g29370* was confirmed. In line 742E06, *At2g38210* was the wrong prediction and *At2g38230* was confirmed.

### Access to the easy-to-use primer design tool

**Figure 2:**
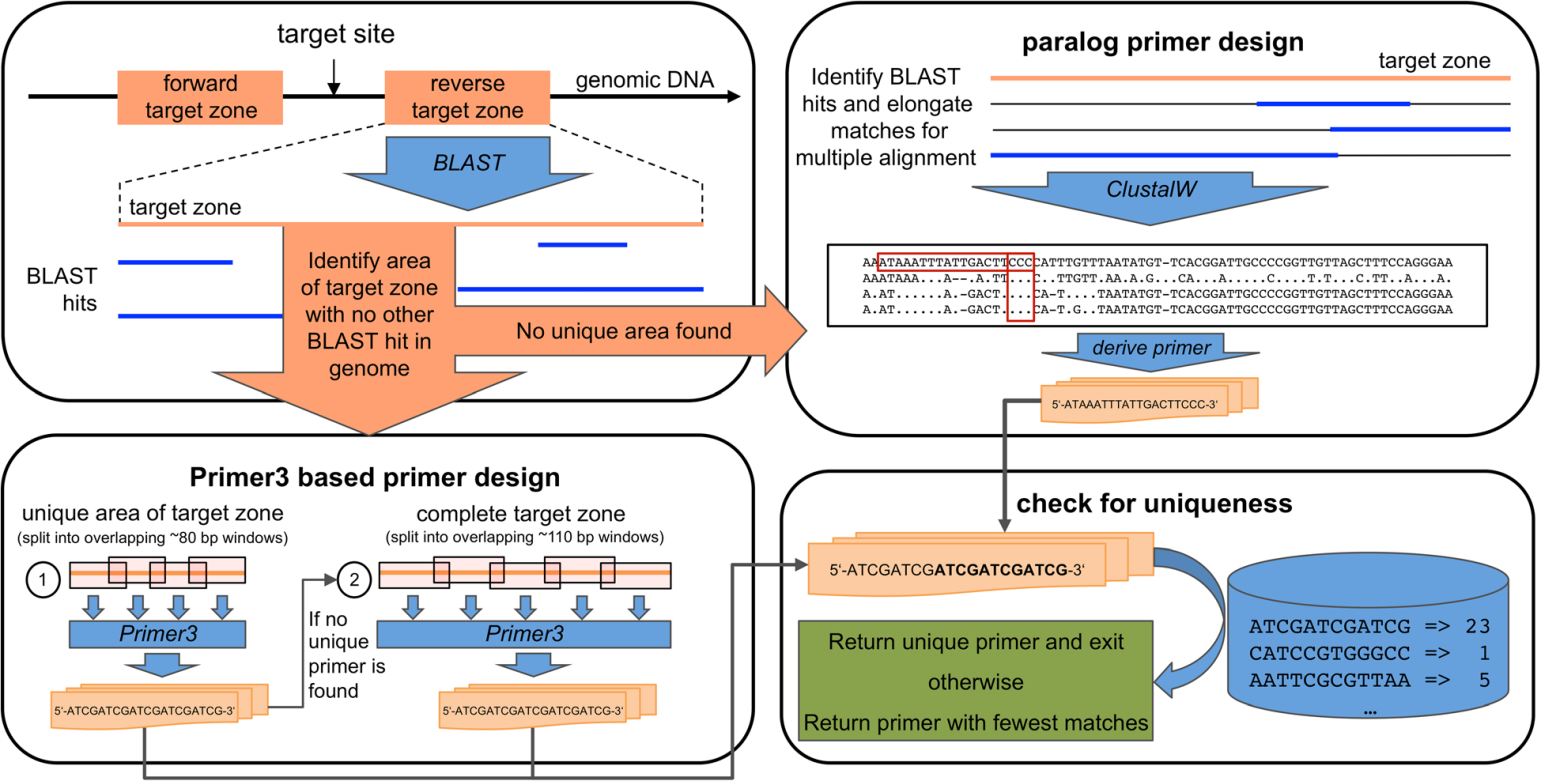
**Overview on the primer design tool.** Initially, each of the two target zones with a distance to the chosen target site (distance and target site defined by the user) is examined by a BLAST vs. the *A. thaliana* genome sequence. If there is an area that has no other BLAST hit somewhere in the genome, the Primer3-based approach is used (bottom left box), otherwise the paralog primer design is used (upper right box). A number of candidate primers is designed in both approaches which are then checked for uniqueness (bottom right box). If a unique primer with no additional 12 bp-hit at the 3′-end in the genome is found, the primer design is stopped and the primer is returned as a result. Otherwise the primer with the fewest matches is returned. When designing additional primers, the next best primers are returned. The Primer3-based primer design (bottom left box) uses multiple runs of Primer3 in overlapping windows and altering temperatures to generate a large set of candidate primers. First, only the unique area of the target zone with no additional BLAST hit in the genome is considered. If no unique primer is found, the process is performed again with the complete target zone. The paralog primer design first creates a multiple alignment with all sequences showing a BLAST hit to the target zone using ClustalW. The algorithm searches for mismatches in the multiple alignment (see Figure 3 for details). To reduce the runtime of ClustalW for sequences with many hits, the target zone is also split into overlapping windows and alignments are computed separately (not shown in Figure). The primer sequences shown in the figure are to be regarded as example sequences that cannot fit to *all* features of the scheduled workflow.

**Figure 3:**
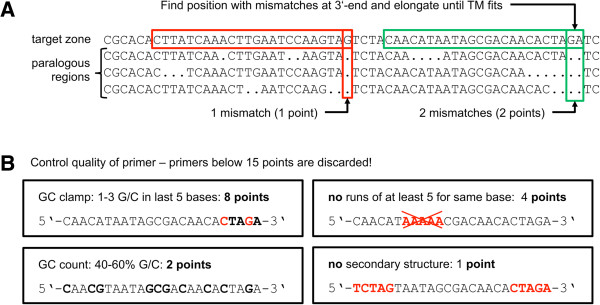
**Paralog primer design. (A)** Possible primers are identified by finding mismatches within a multiple alignment. Each mismatch is worth one point, which is later used to determine the quality of the primer. **(B)** Further criteria are: (i) GC-clamp: there should be 1 to 3 G or C bases in the last 5 bases of the primer (8 points); (ii) primers should not have runs of more than 5 identical bases (4 points); (iii) the GC content should be between 40% and 60% (2 points); (iv) the last 5 bases of the primer should not form a secondary structure with the first 5 bases or rather not be the reverse complement (1 points); all primers need to have at least 15 points in order to be considered as candidate.

**Figure 4:**
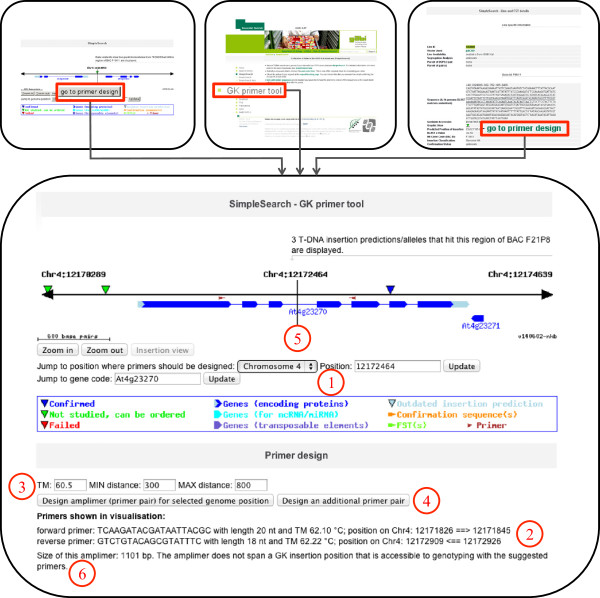
**Integration of the primer design tool in SimpleSearch.** There are three ways to enter the primer design tool in SimpleSearch: (i) via the navigation menu following the link “GK primer design”; (ii) via a link from an insertion prediction (“go to primer design”) that leads directly to the respective insertion position; (iii) via a button in the visualisation (“go to primer design”) for the current central position. The target position for the primer design can be chosen in the user interface by using the chromosomal position according to the TAIRv10 genome sequence (“Jump to position …”), or by using the AGI code of the gene of interest (“Jump to gene code …”) (1); primer sequences are listed at the bottom as well as their positions (2); the annealing temperature and desired distance to the target position can be specified (3); primer design is initiated by using the respective button, after one primer pair has been designed additional primer pairs can be made using the button right to it (4); primer pairs are shown in the visualisation, when more than one primer pair has been computed the one generated last is shown here (5); the amplimer size for the designed primers is shown, as well as possible primer combinations for genotyping, which are accessible via a mouse-over window when GK insertion predictions are spanned by the amplimer (6).

In order to offer public access to the primer design algorithms developed at GABI-Kat, we implemented an easy-to-use tool into SimpleSearch that includes the paralog-specific design if necessary. The design method is chosen as described in Methods and an overview on the primer design process is shown in Figure 2. Details on the selection of suitable primers using the paralog-specific design are summarised in Figure 3. The publicly available primer tool was implemented within the visualisation part and displays the location of the designed primers. It can be accessed directly with the URL [20] or via the menu on the GABI-Kat website [10]. Insertions found in SimpleSearch also provide a link to the primer design for their respective position and a button in the visualisation allows quick access to the primer design for the currently selected position in the genome (Figure 4). It differs from previously available tools for the analysis of paralogous regions in a number of important aspects. Other tools, for example the very useful tool Primer-BLAST [19], checked the redundancy of the combination of both primer annealing sites for the amplimers defined by a primer pair. It also uses MegaBLAST of the complete target zone to the genome sequence of the addressed organism to avoid primer design in redundantly matching parts of the target zone. In contrast to this, our tool checks the 3′-ends of every single primer for redundancy, which is essential for the analysis of T-DNA insertions because in this experimental setup only one genome-specific primer is used. Primer-BLAST uses a BLAST of the complete target zone sequence and optimisation of the BLAST alignments by the Needleman-Wunsch global alignment algorithm to perform a specificity check that deselects primer pairs with amplimers on other targets than the submitted template [19]. Our approach to rank primer candidates uses a precomputed index of all occurrences of 12 bp sequences in the *A. thaliana* genome and considers the last 12 bp of a primer candidate. This 12 bp strech was in our experience the crucial part of the primer. For using the tool, a genomic nucleotide position central to the locus to be addressed (designated “target position”) must be selected. Upon starting the tool, primers are automatically designed around this target position with a default minimal and maximal distance of 300 to 800 bp to the target position on each side. We refer to this sequence range surrounding the target position as “target zone”. The distance to the target position can be changed to values between 100 to 1500 bp with a minimum range on each side of the target position of 100 bp. The larger the target zone, the better are the chances to obtain a unique primer. For the primers, the default annealing temperature has been set to 60.5°C, but it can also be set by the user to a value between 50 and 72°C. The primers can either be used in combination with T-DNA border primers in order to confirm a T-DNA insertion of interest, or simply to create amplicons from the targeted genomic locus. If the automatically designed primers are not acceptable to the user for some reason, the design tool can be executed repeatedly to acquire further primer combinations.

As an additional usability feature, the tool determines and reports the size of the amplimer with respect to the pseudochromosome sequence, and presents a summary of information related to genotyping insertion alleles if a (predicted) insertion site is spanned by the amplimer. The PHP code of the tool is available upon request.

## Conclusions

We describe the primer design procedure that has been used successfully and in large scale for confirmation of T-DNA insertion alleles in the GABI-Kat project. Since 2007 [21] users can access the sequences of experimentally proven confirmation primers for confirmed insertion alleles via SimpleSearch. Now, the primer design procedure established at GABI-Kat has been integrated into the publicly available SimpleSearch interface. The tool can be useful for confirming T-DNA insertion alleles, including those from SALK or other insertion mutant collections. At GABI-Kat, usually only one insertion is confirmed per line. After this first confirmation, the lines are donated to NASC and can further on only be ordered from there. Access to the GABI-Kat primer design tool might therefore help in the analysis of additional insertions, which are listed as predictions in SimpleSearch. Moreover, the tool allows easy design of amplimers for the genotyping of insertion alleles because the amplimers spanning the insertion site differentiate between the wt allele (amplicon produced) and the insertion allele (no amplicon; see [14]). The tool presented in this work differs from the already available tools in several aspects, as discussed above. Also, we simplified the primer design process by providing an easy-to-use user interface, which only requires several mouse clicks and no copy-paste of target sequences. Furthermore, existing tools usually require the definition of several parameters by their users for the design of primers, which is often laborious and confusing for the users, especially when the underlying algorithms are unknown to them. Our tool ensures most convenient primer design, because it only requires the absolute minimum of parameter definition. This is mainly the genome position to be addressed which is easily accessible through GenBank or SimpleSearch or even already known. In addition, the distance to the target position to be considered for primer design and a value for the desired melting temperature of the primers is required. A main advantage is that problems with difficult genomic positions are taken care of automatically. With the new tool we hope to contribute to the simplification of the analysis of T-DNA insertions as well as of other PCR-based applications in *A. thaliana*.

## Methods

Plant growth conditions as well as molecular biological methods used for generation of the data used in this study have been described in [3]. General database aspects have been described in [9,21]. All FSTs generated in GABI-Kat are publicly available through ENA/GenBank and SimpleSearch.

### Terminology

We use the term “amplicon” to refer to the DNA fragment that has been physically formed after PCR and which can be sequenced. The term “amplimer” refers to the theoretical construct, which consists of a primer pair (located on opposite strands and with their 3′-ends directed towards each other) and the corresponding source sequence. For example, one can construct several amplimers addressing a predicted T-DNA insertion site, but only the primer pair that is based on correct predictions and/or assumptions about the configuration of the fusion of T-DNA to genomic DNA of the studied insertion allele will allow successful formation of an amplicon.

### Categorisation of insertion sites

The evaluation of FSTs to predict insertion sites was based on hits (weighted sequence similarities) generated by BLAST [8] and by using the TAIRv10 genome sequence and annotation dataset [22]. Initially, only the best BLAST hit for each FST had been stored [18]. To address paralogous predictions, and also “composite FSTs” that contain sequence parts derived from at least two independent insertion sites, we changed the internal GABI-Kat database to allow storing several insertion site predictions for each single FST. The new predictions were categorised based on the e-value of the BLAST hit. Category 0 is assigned to the prediction deduced from the best BLAST-hit as long as its e-value is below (better than) 1e-3. “Best” hits with larger (less significant) e-values were assigned to category 2. There can only be one best hit for each FST, and this hit is selected by using the top-of-the-list hit in the BLAST output, which results in the prediction of category 0. Note that the best hit might end up at the top position by chance if the respective FST region hits several parts of the genome with identical e-values and scores. If there are additional hits from the same FST with e-values below 1e-3, the deduced predictions are classified as category 1. If different regions of the same FST have hits in different parts of the genome, these regions are handled individually to cover the cases of several insertions being deduced from one composite FST. From each of these regions (and in addition to the single category 0 prediction for the complete FST) a maximum of 3 hits are used to produce predictions of category 1; further BLAST hits are ignored. This restriction is necessary to reduce the amount of lab work caused by FST regions that are not only paralogous but repetitive. For further filtering, the deduced insertion sites (i) need to have a distance of 1000 bp to each other to be considered as a new insertion prediction, if they are closer to each other they are assigned to the same prediction, and (ii) are discarded if the e-value difference to the best hit for one FST region is larger than a factor of 1e10. In general, our subsequent analyses considered only the predictions of categories 0 and 1.

### Definition of paralog groups

For a systematic handling of insertion predictions that hit paralogous regions, we clustered them into groups using a hierarchical clustering approach for all predictions generated for a given GABI-Kat line (obviously this has been done for all lines). Starting with groups containing one prediction each, groups were combined if one of the following conditions hold true for each possible combination of predictions between the two distinct groups: (i) the prediction for different loci was based on the same part of the FST sequence (with a minimal overlap of 30 bp); (ii) the 400 bp of sequence next to the predicted insertion site have an identity of more than 79% to all members of the group. The clustering stopped when no further groups could be combined. All groups that contained at least two predictions for distinct insertions (i.e. they must display more than 1000 bp distance) were stored.

### Primer design avoiding multiple annealing sites for the 3′-end

A primer was regarded as “unique” if its 12 bp-3′-end had only one hit in the genome sequence. The number of occurrences of each 12 bp sequence within the genome has been precomputed and stored in our database. By using this index, the number of possible matching positions for each primer can be identified easily by a simple and fast database query.

We have developed two methods for primer design. One is based on the widely used primer design tool Primer3 [23] with additional filtering, the other uses a self-developed algorithm that searches for mismatches within a multiple alignment of sequence-related target zones. For in-house confirmation at GABI-Kat, primers for insertions that are not located in paralogous regions are designed using the first method, while the second method is applied to insertions in paralogous regions. The public primer design tool works for all positions within the *A. thaliana* genome, is not dependent on (predicted) GABI-Kat insertion sites and automatically chooses the best method for the genomic locus addressed. In order to decide which method is suited best for the target zone in question, a BLAST of the sequences of this zone (that is, the sequences surrounding the target position limited by the value set for the distance to the target position) is performed against the *A. thaliana* genome sequence with an e-value cutoff of 1e-5, which is high enough to detect hits of down to 24 bp. If there is a sequence part within the target zone that has no other hit in the *A. thaliana* genome and is at least 100 bp long, problems with paralogous regions should not occur and the Primer3-based method is used. If such a unique sequence part cannot be determined in the target zone sequence, the paralog primer design method is chosen.

### Primer3-based method

In order to find primer candidates within a target zone, the part of the target zone without additional paralogous regions in the genome (identified during the decision which primer design method should be used) is used first. This part of the target zone sequence is divided into overlapping windows of at least 80 bp and for each of these windows a primer is designed by Primer3. Windows overlap with 30 bp to ensure that possible primers at the ends of the windows are considered as well. To further increase the number of possible primers the selected melting temperature is altered in steps of 0.4°C to maximally 1.2°C below or above the defined melting temperature. All primers are checked for uniqueness of their 3′-end as described above. As soon as a primer is detected that has only one 12 bp-hit within the genome, the primer design finishes successfully. If the initial search within the unique part of the target zone did not yield a result, the procedure is repeated within the whole target zone sequence with a window size of at least 110 bp and alternating melting temperatures in the same way as described above. If no unique primer could be found, the one with the fewest 12 bp-hits among all primers designed during the whole process is regarded as the best possible primer for this target zone.

### Paralog primer method

In target zones that do have paralogous regions throughout their sequence somewhere in the genome, the Primer3-based approach often does not lead to satisfying results. Our algorithm first identifies all potentially paralogous regions within the genome by an initial BLAST with an e-value cutoff of 1e-5 and a minimum required length of 50 bp. All hits are elongated to fit the length of the target zone, and a multiple alignment is computed using ClustalW [24]. In order to reduce the runtime of ClustalW, a sliding window approach with overlapping windows of sizes around 220 bp (and an overlap of 30 bp) is used to compute the multiple alignments. In these multiple alignments, the algorithm searches for positions with a maximum number of mismatches to the sequence of the target zone. This position is defined as the 3′-end of a possible primer and is elongated to match the desired melting temperature. After that, the primer candidate is checked for GC-clamp (1–3 G/C in the last 5 nucleotides), base repeats (less than 5 identical nucleotides in a row), GC-content (between 40 and 60%) and secondary structures (last 5 nucleotides should not appear as reverse complement in the primer). The more of these conditions hold, the better the primer – primers not fulfilling some of these criteria are discarded (see Figure 3). The annealing temperature of the primer is computed using the same formula used in Primer3 (according to [25]) to achieve results comparable to those from the Primer3-based method:

$$T_{m}\left[\mathrm{{}^{\circ}C}\right]=81.5-11.6+0.41\left(\%\mathrm{GC}\right)-\frac{600}{\mathrm{length}}$$

A large number of candidates are generated and further checked for uniqueness as described above. If a unique primer is found it is returned as result. If this is not possible, the primer with the fewest matches among all examined primers is returned.

## Competing interests

The authors declare that they have no competing interests.

## Authors’ contributions

GH, NK and BW conceived and designed research, analysed and interpreted the data and wrote the manuscript. GH conducted wet-lab experiments. NK did database programming and bioinformatics. All authors read and approved the manuscript.

## Authors’ information

Gunnar Huep and Nils Kleinboelting are joint first authors.

## Supplementary Material

Additional file 1: Table S1 The file contains a table (Table S1), which shows statistics about insertion site predictions in the GABI-Kat collection before and after the 1-to-N analysis of the FSTs. An explanation for the data presented in Table S1 is included in the file as well. Click here for file
