# Supplementary material for: An easy-to-use primer design tool to address paralogous loci and T-DNA insertion sites in the genome of Arabidopsis thaliana
Source: Plant Methods. 2014 Sep 13;10:28. doi: 10.1186/1746-4811-10-28 (PMC4169229; doi:10.1186/1746-4811-10-28)
Supplement: Additional file 1: Table S1 — The file contains a table (Table S1), which shows statistics about insertion site predictions in the GABI-Kat collection before and after the 1-to-N analysis of the FSTs. An explanation for the data presented in Table S1 is included in the file as well. [file 1746-4811-10-28-S1.pdf]

Table S1. Statistics for insertion site predictions in the GABI-Kat collection
The table is divided into three parts. First, data for whole FSTs are presented. Second, data from the "1-to-N" FST analyses which allowed to consider more than one BLAST hit per FST are shown. Third, data for paralog groups are summarised. Hits from category 2 were not considered.

	**total numbers**	**only one hit**	**two or more hits**
No. of FSTs with at least one BLAST hit to TAIRv10 genome sequence:	135,210		
No. of predicted insertion loci [1), 2)]:	91,383		
FSTs separated according to No. of hits:		105,399	29,811
No. of deduced insertion site predictions:		105,399	80,210
No. of predicted insertion loci [1)]:		72,202	68,679
No. of FST regions with insertion site prediction:	153,919		
No. of predicted insertion loci from FST regions [1), 2)]:	102,494		
FST regions separated according to No. of hits:		137,985	15,934
No. of deduced insertion site predictions:		137,985	45,028
No. of predicted insertion loci from FST regions [1)]:		94,260	38,038
No. of paralog groups:	10,737		
No. of predicted insertion loci in paralog groups [1)]:	28,836		
No. of FST regions with insertion site prediction in paralog groups:	17,318		
Paralog groups containing loci with very similar likelihood [3)]:	4,605		

[1)] <1000 bp difference for the insertion site prediction in individual lines required
[2)] only best prediction in a given line is counted
[3)] difference in BLAST e-value for predictions of <1e-5 required

Comment to Table S1: GABI-Kat lines usually contain more than one T-DNA insertion, and for most lines several FSTs have been produced. Although the different FSTs from a given line might lead to the prediction of different insertion loci, it is also possible that the different FSTs are derived from a single insertion site. This is the reason for the lower number of "predicted insertion loci" compared to the insertion site predictions according to whole FSTs or regions of FSTs. For the differentiation between the same and independent insertion loci, we empirically determined a distance requirement of 1000 bp for insertion site predictions from a given line. If similar insertion positions are found in different lines, they are counted separately even if the difference of the predictions is less than 1000 bp.

For the definition of different regions of FSTs with hits in the genome we allowed a maximum of 10 bp overlap of the BLAST hits of the regions. The increase of the number of total insertion site or loci predictions when analysing FST regions compared to whole FSTs was explained by the contribution from composite FSTs derived from lines with more than one T-DNA insertion (153,919/102,494 vs. 135,210/91,383).

The table differentiates if one or more predictions have been derived from FSTs or FST regions. It displays in this way the gain in the number of insertion site predictions from the 1-to-N analyses. Concerning the statistics for FSTs or FST regions with more than one paralogous prediction from a single region we allowed a maximum of 1e-10 in the BLAST hit difference. If the difference is even more significant, further paralogous predictions are ignored. The increase from paralogous predictions is most obvious for FST regions with more than one BLAST hit (15,934 FST regions resulting in 45,028 insertion site predictions).

The numbers concerning paralog groups indicates how often the precision of insertion site predictions might be affected by closely related paralogs. The number of 4,605 paralog groups containing locus predictions with a BLAST e-value difference of less than 1e-5 also demonstrates the necessity of an automatic primer design, which takes paralogous regions into account. The "No. of FST regions with insertion site prediction in paralog groups" (17,318) is higher than the total number of FST regions with more than

one hit (15,934) because additional FST regions with only a singe hit were included if they support one of the insertion site predictions included in a paralog group within a given line.
